# Theoretical understanding of water splitting by analyzing nanocatalyst photoabsorption spectra

**DOI:** 10.1515/nanoph-2024-0432

**Published:** 2025-02-03

**Authors:** Prince Gollapalli, Maytal Caspary Toroker

**Affiliations:** Department of Materials Science and Engineering, 26747 Technion – Israel Institute of Technology, Haifa 3200003, Israel; The Nancy and Stephen Grand Technion Energy Program (GTEP), Technion – Israel Institute of Technology, Haifa 3200003, Israel

**Keywords:** nanocatalyst, water splitting, photoabsorption, surface states, absorption spectra

## Abstract

Photons can be used to either monitor or induce catalysis by acting as photoexcited holes or quasi particles, which aid in water splitting reaction leading to a major step towards sustainable energy. However, the mechanism of catalysis using nanocatalysts under photo-illumination is not fully understood because of the complexity involved in three major steps during the oxygen evolution reaction: photoabsorption on nanocatalyst, hole transport to the surface, and the reaction kinetic barriers at the surface. In a photoelectrochemical cell used for water splitting, the surface states of optically and chemically dominant species affect the catalysts’ performance. For instance, the signature of the dominant absorption peak at 580 nm in the observed spectra of Fe_2_O_3_ photoanode can shed light on the oxygen evolution reaction mechanism since each reaction intermediate affects the absorption spectrum, and the absorption coefficient in turn affects the photocurrent. In the recent decade, a combination of different theoretical methods starting from density functional theory up to Bethe–Salpeter equation accounting for excitonic effects helped to establish that the *O intermediate is the rate limiting step in agreement with experimental data. Therefore, this perspective focuses on the complexity and variety of fundamental phenomena involved in water splitting mechanism and various theoretical methods applied to address these and also suggests how the predictive capability of these methods can be used to understand mechanisms beyond water splitting, such as CO_2_ reduction.

## 1 Introduction

Decomposition of water (H_2_O) into its components oxygen (O_2_) and hydrogen (H_2_) gases, widely known as water splitting, can be achieved when visible light absorption occurs on the surface of the catalyst materials such as Fe_2_O_3_, RuO_2_, NiOOH, TiO_2_, WO_3_, perovskite oxides, etc. [1], [2], [3], [4], [5]. When the thickness of these catalyst materials is in nanoscale regime, typically less than 100 nm in at least one of the dimensions, they are referred to as nanocatalysts. Nanocatalysts offer the advantage of high surface-area-to-volume ratio, enabling more catalytically active sites leading to higher rates or turn-over-frequency of O_2_ and H_2_ evolution during water oxidation (on anode) and water reduction (on cathode) reactions, respectively. Understanding these reaction mechanisms gained significant attention because green hydrogen can be produced by photo-illumination under an applied potential without carbon emissions. During water oxidation reaction, the photo-illumination on the anode material generates photoexcited electrons and holes. The holes that are generated could successfully transport to the surface, participate in the water oxidation reaction to generate O_2_. Thus, the photons can activate this catalytic reaction and generate photocurrent. Thus, understanding the photoabsorption spectrum, factors responsible for obtaining this spectrum, and how this spectrum affect the properties such as photocurrent will provide insights into the reaction mechanism. Since this catalytic reaction occur as several reaction intermediates simultaneously on the catalytically active surface, proper choice of catalyst surface facet, and the applied electrical bias beyond the thermodynamic potential (1.23 V) for the rate determining step dictates the overpotential. The shape and size of the nanocatalyst, hot carrier lifetime, occupation of charge carriers on the electronic levels, and chemical stability under alkaline or acidic mediums play a role in visible light absorption during photocatalysis [6], [7], [8]. Proper design of nanostructures can be achieved through doping, alloying, layering, annealing, band gap tuning, and improving charge transport to surface [9], [10], [11].

Since several intermediate reactions are occurring concurrently during photocatalysis, delineating the reaction steps in this light–matter interaction mechanism is important. First, photoabsorption happens when the incident photon of energy *hν* is at least greater than the bandgap of the nanocatalyst. This results in the generation of electron–hole pairs as shown in equation (1).

(1) $$4\;h\nu \to 4\;e^{-}+4\;h^{+}$$

Then, the generated electron–hole pairs are separated, subsequently after fast thermal relaxation, the photo-generated electrons reach the conduction band edge and holes are left at the valence band edge. Together with applied bias, the hole charge carriers are utilized then to promote self-chemical reaction, making all reaction intermediate steps in water oxidation reaction exothermic. The holes transport to the surface of a nanocatalyst and oxidize H_2_O to produce O_2_ in an oxygen evolution reaction (OER) at the anode as shown in equation (2).

(2) $$4\;h^{+}+2\;H_{2}O\to O_{2}+4\;H^{+}$$

Similarly, the electrons transport to the cathodic surface through the external wire and reduces H^+^ to H_2_ resulting in a hydrogen evolution reaction (HER) as shown in equation (3).

(3) $$4\;e^{-}+4\;H^{+}\to 2\;H_{2}$$

The overall reaction is shown in equation (4),

(4) $$2\;H_{2}O+4\;h\nu \to O_{2}+2\;H_{2}$$

**Figure 1:**
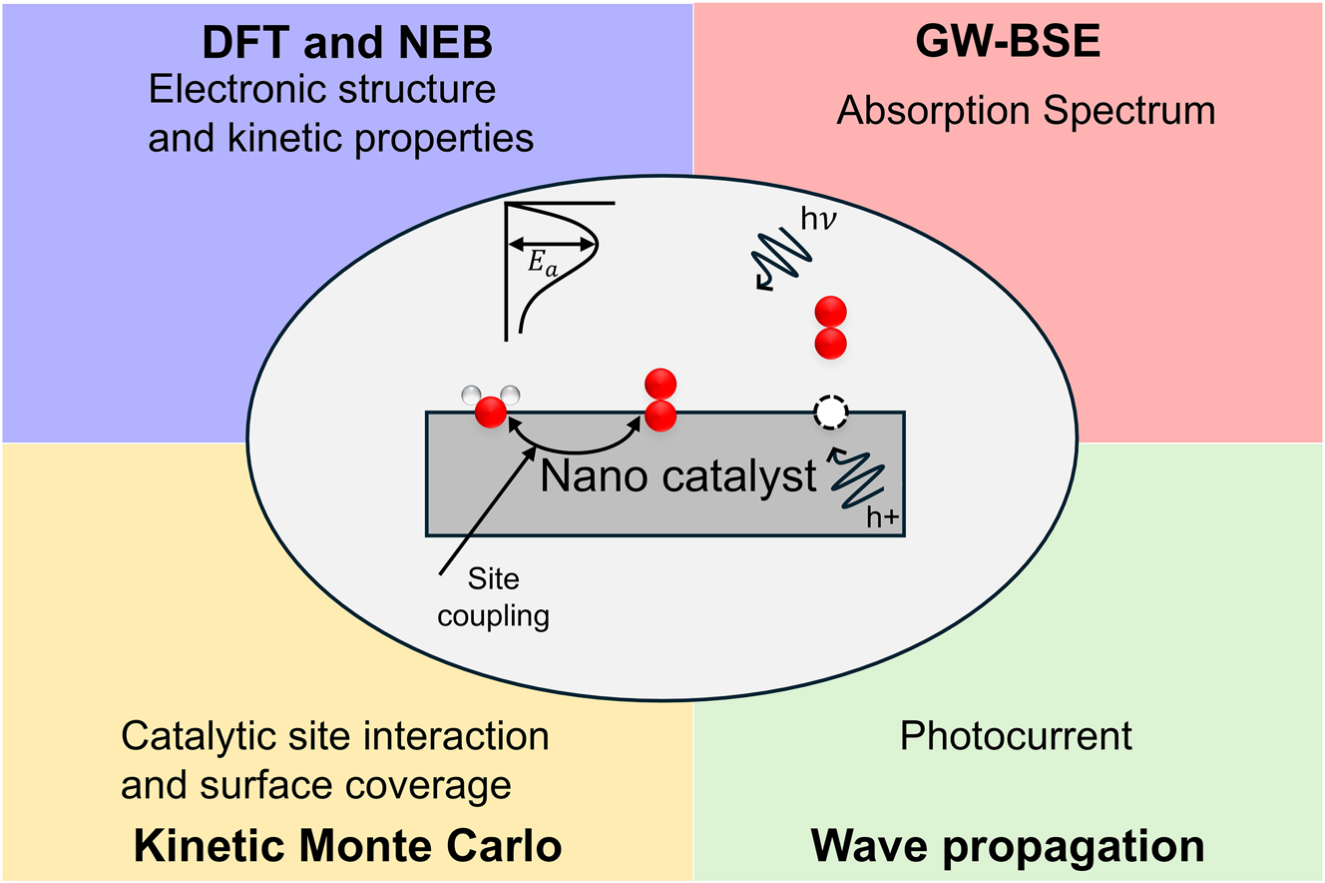
Schematic of several theoretical methods to understand the oxygen evolution reaction (OER) on the surface of a nanocatalyst under photo-illumination. Red and white spheres represent oxygen and hydrogen atoms, respectively.

From equations (1)–(4), we see involvement of light–matter interaction on the surface of a nanocatalyst. A variety of materials were studied as candidate materials for nanocatalysts, such as nanolayers, nanoclusters, cage-like structures, etc. . Here, we highlight some of these materials, but as a test case, we primarily focus on crystalline hematite (*α*-Fe_2_O_3_) as a nanocatalyst and anode material that performs the half-cell water oxidation reaction. During the oxygen evolution reaction (OER) on the Fe_2_O_3_ surface, the measured photoabsorption spectrum showed a dominant absorption peak at 580 nm. There has been a long-term debate on origin of the 580 nm peak whether it is a result of a peroxo chemical species intermediate (the *Fe–O intermediate) or a surface defect. By considering several possible adsorbates, DFT + U was helpful in answering that *Fe–O intermediate corresponds to the 580 nm peak [17]. Since this reaction always involved four electrons and four holes, a successful attempt was to consider four step reaction pathway mechanism involving one proton-coupled electron transfer (PCET) [18] in each step. A schematic description of equations (1) and (2) and several theoretical methods used to understand the oxygen evolution reaction mechanism is shown in Figure 1. The absorption spectra measured operando is a demonstration of light–matter interaction that can help detect the most dominant surface species responsible for rate limiting step of the reaction.

Although Fe_2_O_3_ requires a large overpotential for water oxidation catalysis and low electronic conductivity (large electron–hole recombination rate), several approaches were adopted to overcome these challenges. Doping affects the overpotential [19], [20] of Fe_2_O_3_: doping with transition elements such as cobalt reduced the OER overpotential by 0.16 V [21]. Additional strategies for controlling overpotential include oxide underlayers and overlayers .

Since Fe_2_O_3_ is an earth-abundant oxide that has been highly studied for improving water splitting, this material serves as a good candidate for benchmarking and analysis. Understanding the OER reaction and the absorption spectra is a complex process because it involves calculating several variables: Gibbs free energy of the rate limiting step and other reaction intermediates, the activation energy required to overcome the kinetic barrier, exciton oscillator strength, dielectric constant, charge transport, etc. Moreover, the interaction with light needs to be accounted for in photocatalysis. The photonic effects are sensitive to the crystal structure and its bandgap as well. Electronic structure calculation complements the understanding of OER reaction mechanism where proton transfer and chemisorption kinetics during intermediate reactions in picosecond time domain can be simulated [25]. Indeed, a variety of theoretical methods were used to provide a holistic view of the water splitting mechanism due to photo-illumination.

Herein, we provide a perspective on various computational modeling approaches used to resolve the chemical origin of the photogenerated absorption peak observed in OER and as a consequence identify the rate limiting reaction step using Fe_2_O_3_ as a catalyst. The theoretical methods used to help explain experimental findings include density functional theory, GW-Bethe–Salpeter equation (BSE), kinetic Monte Carlo, nudged elastic band method, and wavefunction propagation. Density functional theory helped in understanding the reaction limiting step from the Gibbs free energy changes of reaction intermediates, then using GW-BSE, which takes excited states into account, helped generate absorption spectrum for the intermediate reactions, and the results showed a match between the calculated and experimental absorption spectrum at the spectral peak locations [26], [27]. Kinetic Monte Carlo techniques were used to study the surface coverage. A combination of Marcus theory with the climbing image nudged elastic band method helped identify the kinetic barriers for the reaction. Wave propagation approach helped identify the current density–voltage (J–V) characteristics under dark current and photocurrent. Also, we expand our discussion on how these theoretical methods were used to uncover the reaction mechanisms involved in CO_2_ reduction by analyzing the absorption spectra of the catalyst. This perspective is divided into four sections: (1) what are the atomistic mechanisms contribute to the optical absorption spectrum, (2) studying the absorption spectrum, (3) how does the absorption coefficient affect the photocurrent, and finally (4) how these computational methods can be used to understand CO_2_ reduction reaction.

## 2 Density functional theory (DFT) as electronic structure initial input for optical properties

The optical absorption of the nanocatalyst will be affected by its electronic structure. Although DFT is a ground-state method that is helpful in obtaining ground state geometry and properties, this information can be used as an input to the GW-BSE calculation to calculate optical properties. To understand this effect theoretically, first the crystal surface needs to be considered. Then during the process, the surface coverage and absorption spectra of various reaction intermediates on this surface need to be evaluated. As a test case, we are focusing on the OER reaction on the (0001) surface of *α*-Fe_2_O_3_ (from now on referred as Fe_2_O_3_). This Fe_2_O_3_ can be considered as a nanocatalyst because the (0001) surface is modeled with a height of ∼ 1 nm and other two directions are periodic. Experimentally, Fe_2_O_3_ as a photoanode for OER reaction was studied under alkaline conditions owing to its stability in strongly alkaline solution, and also as an abundant and inexpensive visible-light absorber [28], [29]. Thus, the DFT studies that calculate the surface electronic structure and reactivity mostly considered strongly alkaline condition of 13.6 pH. Thus, equation (2) is valid only under acidic condition, whereas under basic condition, equation (2) will be translated into equation (5).

(5) $$4\;h^{+}+4\;OH^{-}\to O_{2}+2\;H_{2}O$$

**Figure 2:**
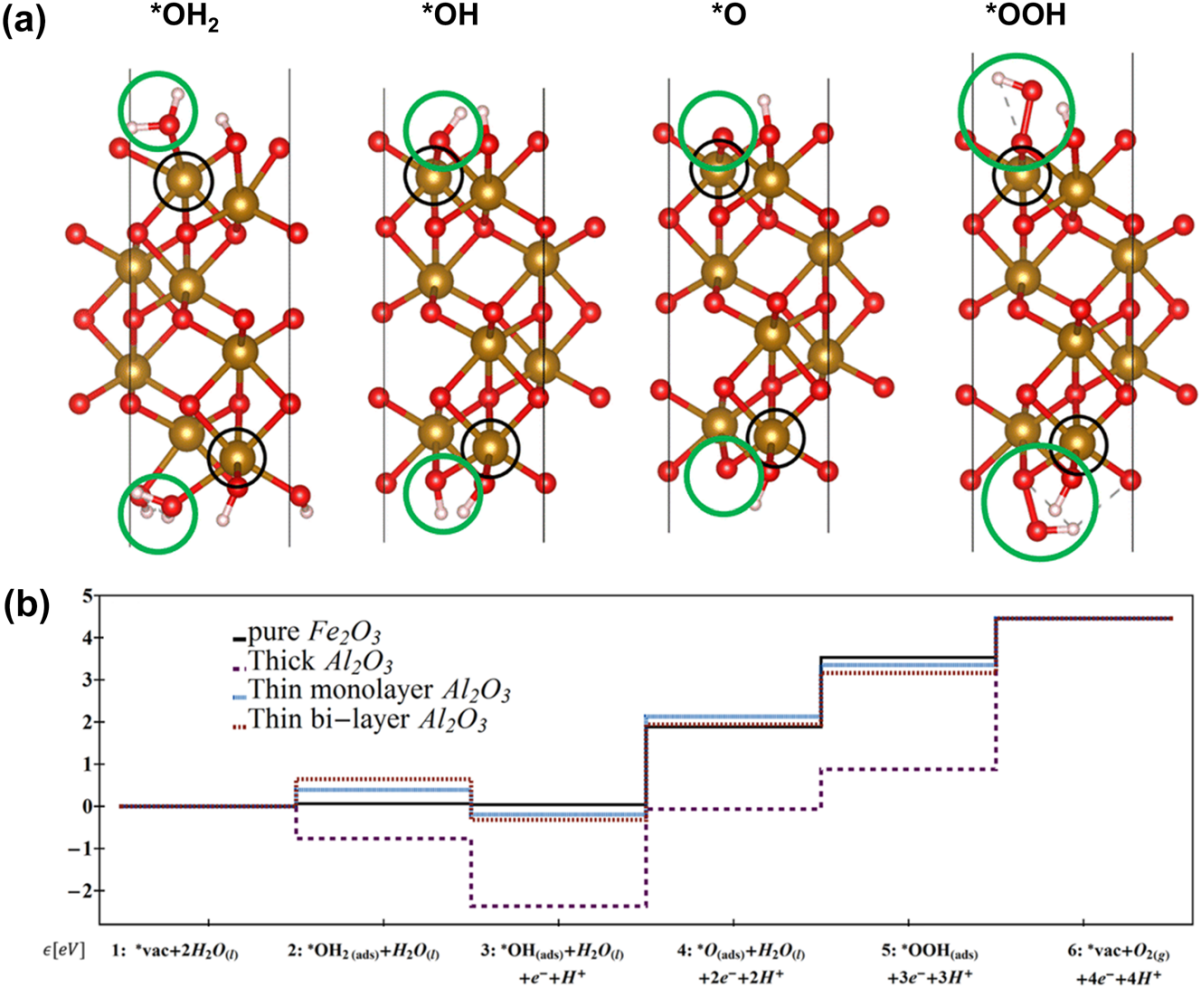
(a) (0001) surface of *α*-Fe_2_O_3_ on which splitting of water into O_2_ occurs, i.e., water oxidation/oxygen evolution reaction (OER) occurs via reaction intermediates OH_2_, OH, O, and OOH. These adsorbates are shown in green circles, and the active Fe site on which OER reaction occur is indicated in black circle. (b) Cumulative Gibbs free energy changes during transition process of reaction intermediates, starting from initial H_2_O absorption to final release of O_2_ gas. Adapted Fig. (a) with permission from [38]. Copyright 2024, Royal Society of Chemistry, CC BY-NC 3.0 Unported Licence. Reprinted Fig. (b) with permission from [31]. Copyright 2015, American Chemical Society.

Since this reaction takes place on Fe_2_O_3_ surface, the surface with H_2_O adsorption under a basic (OH^−^) environment needs to be modeled for each reaction intermediate. The highest activation energy for the reaction intermediate corresponds to the slowest step, i.e., the potential determining step (PDS), and the surface will be dominantly covered by this reaction intermediate and will have the most effect on the photoabsorption spectrum. The individual intermediate reactions of the equation (5) are shown in equations (6)–(10), and their crystal surfaces are shown in Figure 2a.

(6) $$*+H_{2}O\to *OH_{2}$$

(7) $$*{OH}_{2}+h^{+}+OH^{-}\to *\text{OH}+H_{2}O$$

(8) $$*OH+h^{+}+OH^{-}\to *\text{O}+H_{2}O$$

(9) $$*O+h^{+}+OH^{-}\to *OOH$$

(10) $$*OOH+h^{+}+OH^{-}\to O_{2}+H_{2}O+*$$

In this five-step process, initially, a water molecule adsorbs on the surface of Fe_2_O_3_ nanocatalyst, which is represented in equation (6). Here, * represents Fe_2_O_3_ (0001) surface with a vacant “O” site on its top surface layer, and *OH_2_ denotes an adsorbed H_2_O molecule on the Fe_2_O_3_ surface where the “O” of H_2_O sits in the vacant “O” site on the top surface layer of Fe_2_O_3_. Similarly *OH, *O, and *OOH correspond to the adsorbates of OH, O, and OOH. In the second and third reactions in equations (7) and (8), OH^−^ from the alkaline electrolyte solution and a surface hole (h^+^) of Fe_2_O_3_ interact with the adsorbed species to create a H_2_O molecule and an intermediate with one less H (thus creating deprotonation). In the fourth reaction in equation (9), the OH^−^ adsorbs on the *O intermediate to form the *OOH intermediate. Finally, in the fifth reaction shown in equation (10), the OH^−^ and h^+^ interact with the *OOH intermediate to form a H_2_O molecule and an O_2_ molecule. Once the O_2_ molecule leaves the surface, a vacant “O” site is left behind on the top surface layer of Fe_2_O_3_ (i.e., *), thus enabling the catalytic cycle to repeat.

The Gibbs free energy change (Δ*G*) for these individual reactions can be calculated using density functional theory (DFT+U) with an effective U-J assigned for Fe because of it being a transition metal with localized d orbitals [30]. The value of Δ*G* for the second step is shown as an example in equation (11), and Δ*G* values are plotted for various reaction intermediates as shown in Figure 2b [31].

(11) $$\begin{array}{ll} \Delta G_{2} & =E\left(*\text{OH}\right)-E\left(*{\text{OH}}_{2}\right)+\frac{1}{2}\text{E}\left({\text{H}}_{2}\right)+\Delta \text{ZPE}\\ & \;-T\Delta S-0.0592pH-eU \end{array}$$

Where E is the relaxed-structure’s total energy as calculated by DFT, ZPE is the zero-point energy, T is the absolute temperature, S is the entropy, and U is the external bias relative to the reference hydrogen electrode (RHE). The energy of hydrogen represents the proton that leaves *OH_2_ in the reaction. Similar to equation (11), the free energy differences for all five reactions were calculated without bias. The values of Δ*G* for these five reaction intermediates shown in equations (6)–(10) without pH (with pH=13.6) are 0.05 (0.09) eV, −0.03 (−0.84) eV, 1.82 (1.05) eV, 1.68 (0.84) eV, and 0.91 (0.08) eV [16], [32]. This indicates that *OH to *O and *O to *OOH are the key steps where the values of Δ*G* are the highest. Without including pH, the summation of Δ*G* values for these five reactions when divided by four electrons involved in the process will give 1.11 V, and the experimental value is 1.23 V. For the catalytic reaction to get activated, an overpotential is required that increases the onset potential to activate OER process above this calculated 1.11 V. The overpotential of the reaction pathway is defined as the minimum potential required to make all reaction steps exothermic. Based on the calculations of the Gibbs free energy of each reaction step, the theoretical overpotential *η* was calculated according to equation (12).

(12) $$\eta =\max\left(\Delta G_{1},\Delta G_{2},\Delta G_{3},\Delta G_{4},\Delta G_{5}\right)-V_{\text{OER}}$$

An overpotential of 0.71 V is required to activate the catalytic reaction with Fe_2_O_3_ [32]. An optical absorption spectrum can be generated for each of these reaction intermediates to observe the correlation between the reaction intermediate with the highest Δ*G* and the absorption coefficient.

## 3 Optical absorption

### 3.1 GW-BSE approach to generate optical absorption spectra

**Figure 3:**
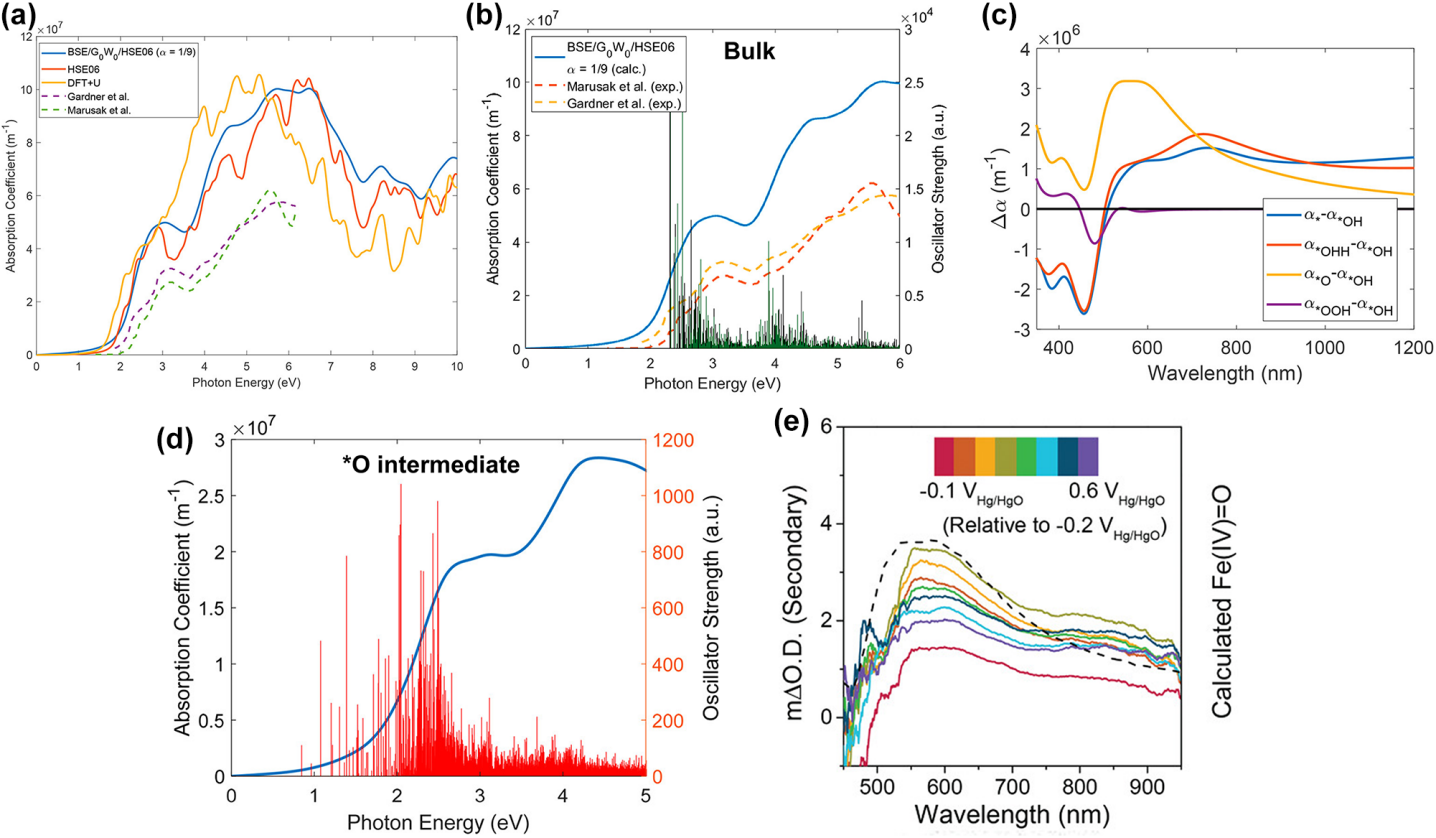
Optical absorption spectrum of *α*-Fe_2_O_3_. (a) The bulk absorption spectra of Fe_2_O_3_ generated from several theoretical methods and comparison with experiment, (b) bulk absorption spectrum and oscillator strength from theory and experiments, (c) difference in absorption coefficient between *OH and other reaction intermediates *, *OH_2_, *O, and *OOH, (d) absorption coefficient of *O intermediate and its oscillator strength, (e) difference in absorption coefficient at different potentials. Reprinted Fig. (a–d) with permission from [26]. Copyright 2020, American Chemical Society. Reprinted Fig. (e) with permission from [27]. Copyright 2021, Wiley-VCH GmbH.

Since optical absorption is an excited state property, and DFT+U based approach considers only ground state phenomenon, it cannot represent excited state phenomenon. Thus, we need to move beyond DFT, such as Green’s function (G) formalism with the screened Coulomb interaction (W) – GW approach to calculate the quasi particle energies. When GW is followed by Bethe–Salpeter equation (BSE) [33], the exciton (e^−^–h^+^ pair) energy and the oscillator strength of the excitons can be obtained. BSE considers excitonic effects into its dielectric tensor calculations, making it one of the most accurate methods to calculate the absorption spectrum. Although this method is computationally more demanding than the DFT, it enables obtaining the peak locations in the spectrum accurately as can be seen from Figure 3a [26]. The absorption coefficient of the nanocatalyst is related to its dielectric constant as shown in equation (13).

(13) $$\alpha =\frac{4\pi }{\lambda }\sqrt{\frac{-\varepsilon_{r}+\sqrt{\varepsilon_{r}^{2}+\varepsilon_{i}^{2}}}{2}}$$

Where *α* is the absorption coefficient, *λ* is the wavelength, and *ɛ*_*r*_ and *ɛ*_*i*_ are the real and imaginary parts of the dielectric constant. Figure 3b shows the absorption coefficient of the bulk Fe_2_O_3_ when averaged over all three axes. The green bars in Figure 3b represent the oscillator strength of excitons starting around 2.3 eV since the absorption starts when the energy of incident photon *hν* is at least higher than the bandgap of the material (bandgap of Fe_2_O_3_ is around 2.2 eV [34]). The oscillator strength depends on the exciton binding energy, which is correlated to the photon energy according to equation (14).

(14) $$h\nu =E_{g}-\frac{E_{X}}{n^{2}}$$

Where *hν* is the photon energy, E_g_ is band-to-band optical gap obtained from BSE, E_X_ the exciton binding energy, and n is the excitation order. From the difference in BSE band gap of the *O intermediate of 2.12 eV and quasi-particle band gap of 2.18 eV (obtained from G_0_W_0_), the exciton binding energy was calculated to be 60 meV [26]. Since these excitons arise from addition of surface species, they behave as defects and bound excitons, which eventually impede holes from leaving the surface due to recombination.

Since reaction happens on the surface of the nanocatalyst rather than bulk, the absorption on individual reaction intermediates provide valuable insight into the reaction. The difference in absorption coefficient between *OH and other surfaces (*, *OH_2_, *O, *OOH) is shown in Figure 3c. The Gibbs free energy change is highest for the *OH to *O transition and also the highest difference in absorption coefficient is between *OH and *O (α_*O_ − α_*OH_). The *O intermediate species shows a midgap state of 0.4 eV above the valence band [17] while others do not. This indicates that the amount of absorption will be most affected by these midgap states. *O shows oscillator strength of excitons even below its bandgap as shown in Figure 3d. Also other intermediates show bright excitons below their bandgap. This points out that the geometrical reconstruction due to bond length difference between the active site and O atom on the surface due to adsorbates generates midgap states, changes oxidation state of the active site, and influences the absorption spectrum. The calculated absorption spectrum at different potentials is compared with the experimental absorption coefficient in Figure 3e. The computational spectrum calculated for the difference in absorption for species Fe(IV)=O and Fe(III)–OH is shown in black dotted line. Even with increasing bias, the peak location in the experimental spectrum is around 580 nm and they do not shift [27], showing good agreement between the experimental and the computational spectrum.

### 3.2 Time-dependent DFT (TD-DFT) approach to generate optical absorption spectra

**Figure 4:**
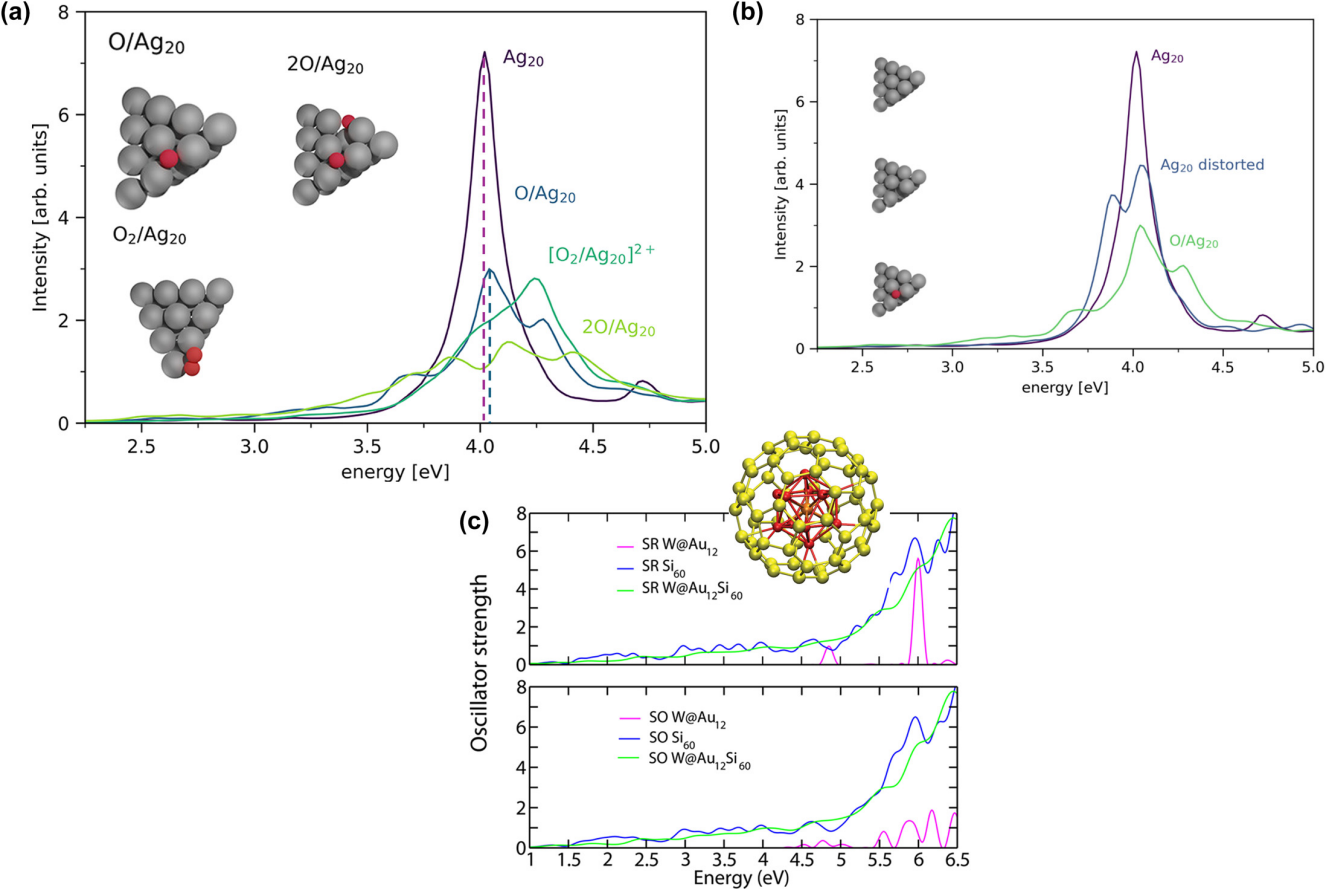
Absorption spectra of (a) oxygen adsorption on Ag_20_ cluster, (b) O-induced geometry of Ag_20_ cluster showing peak split, (c) oscillator strength of cage-like structure of W@Au_12_ and W@Au_12_Si_60_. Adapted Fig. (a–b) from [13]. Copyright 2023, American Chemical Society, CC-BY 4.0. Reprinted Fig. (c) with permission from [12]. Copyright 2013, American Chemical Society.

Apart from the GW-BSE approach, another widely used method to calculate photoabsorption spectra and oscillator strength is time-dependent density functional theory (TD-DFT). The polTDDFT method was used by Zerbato et al. [13] to obtain absorption spectra when oxygen is adsorbed on a silver Ag_20_ cluster. They observed a difference in the peak position between the adsorption of a single O atom, O_2_ molecule, and 2 individual O atoms as shown in Figure 4a. When the absorption spectrum of the Ag_20_ cluster is compared with the same Ag_20_ cluster but with the geometry adapted due to single O adsorption (after removing O), the absorption spectra showed a peak split into two less intense peaks and an overall decrease in the intensity as shown in Figure 4b. This peak split is attributed to the loss of symmetry caused by O-induced Ag_20_ reconstruction. The decrease in intensity was ascribed to the changes in oxidation of the Ag atoms in the vicinity of the adsorbed O atom. When this absorption spectrum is compared with Figure 3d for different reaction intermediates on Fe_2_O_3_ surface, the differences in absorption coefficient can be related to the local geometry changes on the surface and oxidation state. The surface geometry variation due to different adsorbates had an influence on the absorption spectra for each surface reaction intermediate.

When the size of the cluster decreases even further, for instance, 12 atoms of Au, the oscillator strength is affected by spin–orbit coupling [12]. Oscillator strength obtained using TD-DFT for W@Au_12_ and W@Au_12_Si_60_ clusters under scalar relativistic (SR) and spin–orbit coupling (SOC) showed a difference [12] as shown in Figure 4c. The W@Au_12_ cluster is more affected by the SOC as expressed by the splitting of the spectral lines, red shift in energy, and oscillator strength redistribution. Thus, while modeling OER reaction on nanocatalysts, when the material size is very small, spin orbit coupling can affect the optical absorption spectra. Thus, the geometry and size factors affect photoabsorption spectra. Although TD-DFT method captures the electronic level insights in a relatively shorter time scale of a femtosecond compared to the actual chemical reactions causing geometric reconstruction at the surface during several reaction intermediates, it can be still useful to capture underlying electronic changes. After photoabsorption, the next step is holes coming to the surface of the nanocatalyst, and there is an energy barrier involved in this. Once the barrier is crossed, then the catalytic oxidation reaction will be activated.

## 4 Catalytic mechanism under photon illumination using kinetic Monte Carlo approach

The activation energy dictates the reaction rate, which in turn controls the variation in current density with bias. First, we discuss the nudged elastic band (NEB) method to calculate the activation energy, next we show how current density varies with bias using two methods: (1) kinetic Monte Carlo (KMC) approach for dark current and (2) wave propagation approach to include photocurrent.

### 4.1 Kinetic barrier estimation using the nudged elastic band method

The activation of catalytic oxidation reaction results in the generation of O_2_ molecules. The kinetic barrier associated with one of the intermediate steps determines the O_2_ evolution rate. Marcus theory combined with the climbing image nudge elastic method (CI-NEB) was used to find the transition state and activation energies of OER steps [35]. Equation (10) was split into two reaction steps shown in equations (15) and (16) to model the activation energy for the associated barrier.

(15) $$*OOH+h^{+}+OH^{-}\to *OO+H_{2}O$$

(16) $$*OO\to O_{2}+*$$

By combining activation energies and calculated free energy differences, reorganization energy was calculated according to Marcus theory.

(17) $$E_{a}=\frac{{\left(\Delta G+\lambda \right)}^{2}}{4\lambda }$$

**Figure 5:**
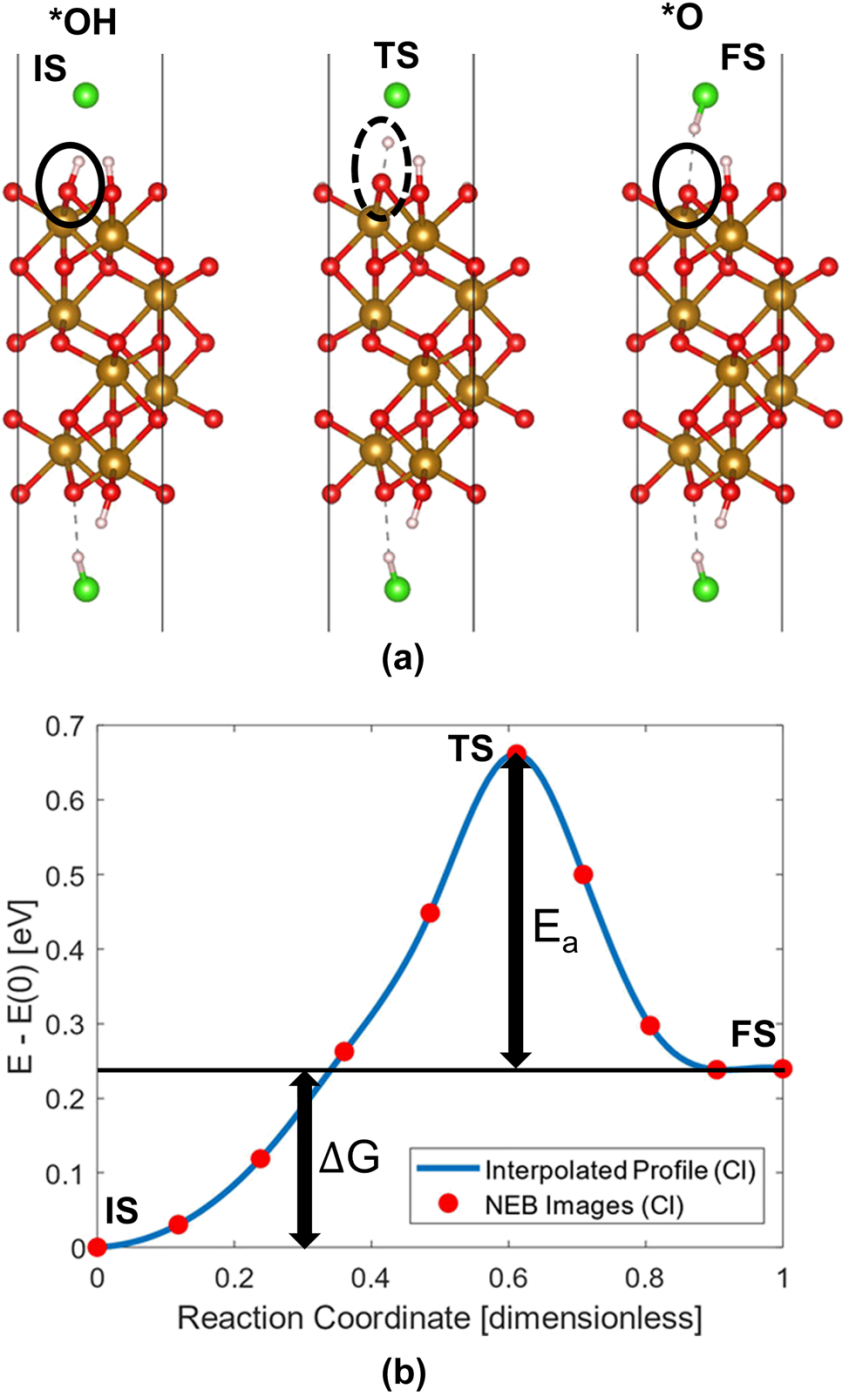
Reaction kinetic barrier estimation. (a) Deprotonation step showing the transition of *OH reaction intermediate to *O. The initial state (IS), transition state (TS), and final state (TS). Red, gold, white, and green spheres represent oxygen, iron, hydrogen, and chlorine atoms, respectively. (b) The thermodynamic barrier is represented as Gibbs free energy (Δ*G*), and kinetic barrier, i.e., activation energy (E_a_) was calculated from the climbing image nudged elastic band method. Adapted from [35]. Copyright 2023, Wiley-VCH GmbH, CC-BY 4.0.

With several activation energies (*E*_*a*_) and free energy differences (Δ*G*) of the same reaction using different ionic “traps” for the deprotonated hydrogen calculated using CI-NEB, the best fit for the reorganization energy, *λ* was obtained. The reorganization energy is then used to calculate the activation energy for different external biases for reactions shown in equations (7)–(10). This algorithm is efficient and simple to use in order to obtain the inner sphere contribution of the reorganization energy to the activation energy. Deprotonation of *OH intermediate to form *O is shown in Figure 5a, and NEB profile of the reaction is shown in Figure 5b. In the initial state (IS), *OH intermediate can be seen. At the transition state (TS), the H atom is located between *O and Cl. And at the final state (FS), *O intermediate can be seen on the surface and H atom is captured by Cl, which is used as a ionic “trap”/sink for H^+^ capture. At pH=13.6 and at 0 V, the rate determining step (RDS) is *O to *OOH reaction having the highest activation energy of 1.277 eV [35], which agrees with other reports including free energy calculations indicating *O is the limiting step of OER with Fe_2_O_3_ [20]. The activation energy decreased from 1.277 V to 0.328 V when the bias was increased from 0 V to 2 V [35]. Since the barrier is decreased, the applied bias will affect the current density.

### 4.2 Kinetic Monte Carlo method to calculate variation in current density under dark light

The values of activation energy *E*_*a*_, obtained from equation (17) can be used to calculate the reaction rate as shown in equation (18) as calculated for the oxygen evolution reaction free energies and the neighboring surface adsorbate distribution during intermediate reactions using a combination of DFT and Monte Carlo simulations to cover the water-front using residence time algorithm (RTA).

(18) $$k=k_{0}\;{\text{e}}^{-\frac{E_{a}}{k_{B}T}}$$

**Figure 6:**
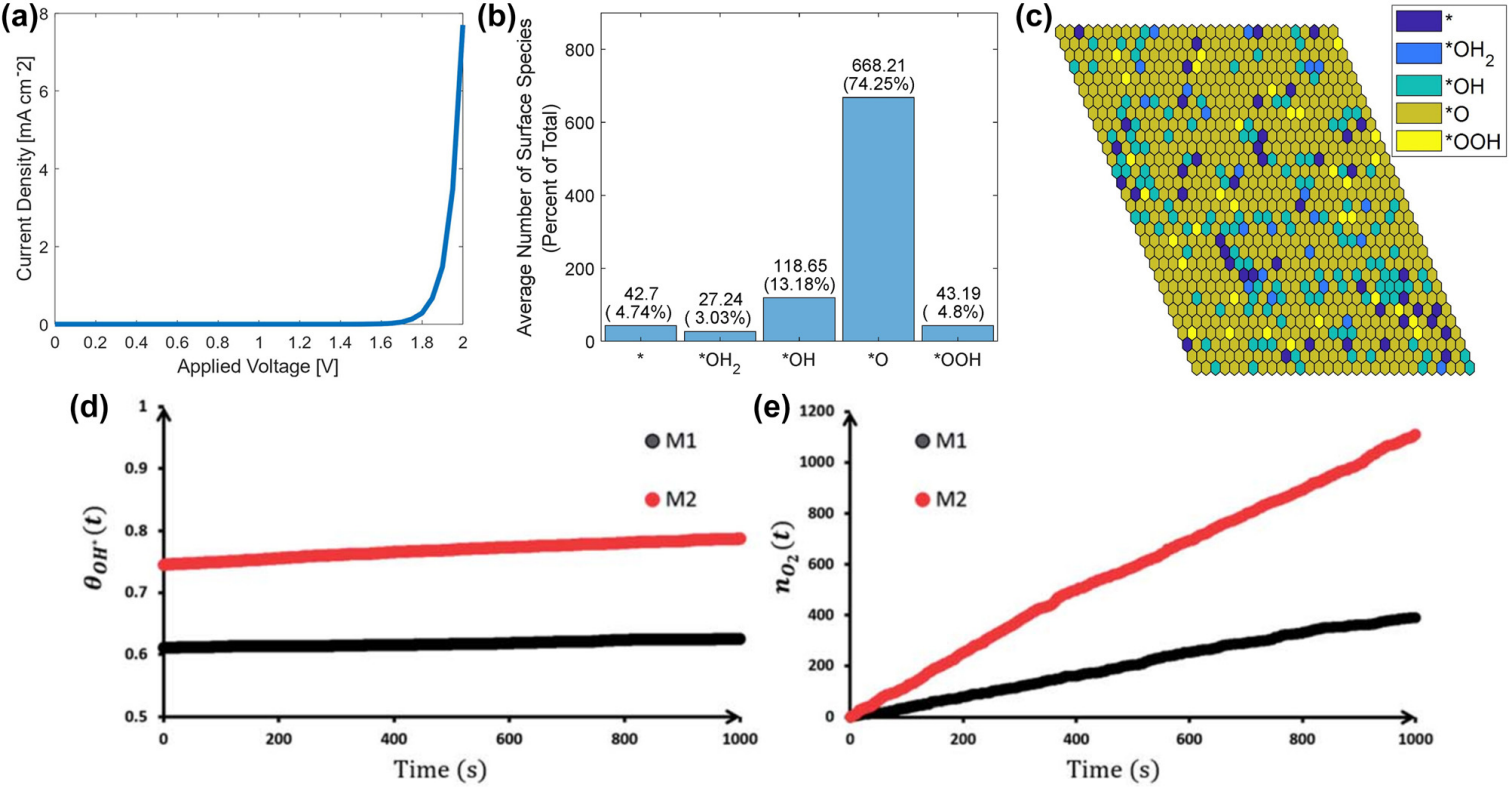
Kinetic Monte Carlo approach to predict surface characteristics. (a) Current density versus applied voltage generated using kinetic Monte Carlo simulation from kinetic barriers obtained from the nudged elastic band (NEB) method, (b–c) surface coverage by reaction intermediates, (d) surface coverage of *OH intermediate, and (e) the oxygen evolution rate predicted from two OER mechanisms, M1 and M2. Adapted Fig. (a) from [35]. Copyright 2023, Wiley-VCH GmbH, CC-BY 4.0. Adapted Fig. (b–c) from [16]. Copyright 2022, Wiley-VCH GmbH, CC-BY 4.0. Reproduced Fig. (d–e) with permission from [37]. Copyright 2021, The Royal Society of Chemistry.

Where *k*_0_ is the *k*_*B*_ *T*/*h* from the transition state theory. Now the total time required for the reaction was calculated by summing up the individual reaction rates [16]. Considering the amount of charges transferred during the simulation per unit area of the surface of Fe_2_O_3_, dividing the charges by total time can provide current density (J) as a function of applied bias (V) as shown in Figure 6a.

Surface coverage can be computed using DFT in combination with kinetic Monte Carlo (KMC) simulation. At several sites on the catalyst surface, reaction intermediates may form simultaneously, and the accessibility of active sites can enhance photoabsorption [36]. Thus, the interactions between reaction intermediates can play an important role in the O_2_ evolution rate. To understand the interactions between catalytic sites, OER free energies and surface adsorption distribution needs to be evaluated. Since *O is identified as the rate limiting step, it is important to understand when the neighboring atoms are other absorbates. This helped to understand that the surface of the catalyst is mostly covered with *O intermediate as shown in Figure 6b and c. *O being most abundant on the surface has high activation energy and inhibits transition to *OOH. This helps us to reinstate that *O intermediate is the bottleneck for OER. The anodic current density is calculated according to equation (19).

(19) $$j_{a}=j_{0}\exp\left(\frac{\alpha nq}{k_{B}T}\eta \right)$$

Where *α* is charge transfer coefficient, n is the number of electrons involved that is equal to 4, q is the electron charge, and *η* is the overpotential.

The current density vs. bias plot (Figure 6a) shows that the onset potential is around 1.8 V; this points out that theoretically 1.11 V from the Gibbs free energy and 0.71 V overpotential together contributing to 1.8 V. Above this 1.8 V, catalysis mechanism will be activated and O_2_ evolution begins. The amount of O_2_ released per unit time is calculated by Sinha et al. [37] on Fe_2_O_3_ (110) surface using microkinetic modeling approach by considering two mechanisms. The mechanism M1 is the same five-step mechanism shown here in equations (6)–(10), and mechanism M2 combines first two steps shown in equations (6) and (7) into a single step as shown in equation (20).

(20) $$H_{2}O+*\to OH^{*}+{\text{H}}^{+}+e^{-}$$

The surface coverage of OH* intermediate and oxygen evolution rate at a given time $\theta_{OH^{*}}\left(t\right)$ and $n_{O_{2}}\left(t\right)$ is simulated for 1000 s at 1.23 V versus CHE (computational hydrogen electrode) as shown in Figure 6d and e. At voltages greater than 1.3 V, the surface coverage of OH* between both the mechanisms are identical [37] because OH* is not the rate limiting step. This points out that the rate limiting intermediate species influence the kinetic barrier. At higher voltages, the kinetic barrier for the rate limiting step decreases, affecting surface coverage and oxygen evolution rate.

Surface coverage also influences the current density [36]. Although current density–voltage (J–V) characteristics can be obtained using the KMC approach under dark current, it is also important to calculate the current density under photo-illumination giving rise to photocurrent, which depends on the hole flux reaching the surface.

### 4.3 Wave propagation method to calculate photocurrent

The wavepacket propagation helps understand the hole transport to the surface. The photocurrent generated is directly proportional to the optical absorption coefficient [38], [39]. Zhao et al. [40] showed that surface plasmon resonance (SPR) coupled with a built-in electric field originating from piezoelectric ZnO can affect the absorption spectra. Increased absorption of visible light promotes charge transfer and separation of photogenerated electrons/holes. The amount of photocurrent generated depends on the fraction of holes that could successfully transport to the surface without being recombined. A wave propagation method for charge transport across materials was developed and used to predict photocurrent [41], [42]. To calculate photocurrent, the electron flux (speed of electron at each time step) has to be summed up over time. It is to note that the wave propagation method, although it finds the solution of Schrödinger equation at each time step by propagating the wave, is not same as time-dependent DFT because the algorithm is in fact a solution to time-dependent Schrodinger equation with a potential energy, initial momentum, and initial position that are obtained from a time-independent DFT calculation. From output of time-independent DFT, the initial momentum, i.e., conduction band minima, can be translated as initial kinetic energy, and also the initial position of electrons can be represented using a Gaussian function shown in equation (21).

(21) $$\psi_{0}=\psi \left(x,0\right)=Ae^{-\frac{{\left(x-x_{0}\right)}^{2}}{2\sigma^{2}}}{\text{e}}^{\text{i}k_{0}x}$$

For each intermediate step shown in equations (7)–(10), proton (H^+^) leaves the surface involving a hole (and a OH^−^ ion) that perform transport [38]. To understand the hole transfer dynamical contribution, one-dimensional time-dependent Schrödinger equation is used to propagate the wave, by considering position space wavefunction $\psi \left(x,t\right)$ and advancing it by a small time step Δt as shown in equation (22), derived according to reference [38].

(22) $$\psi \left(x,t+\Delta t\right)=e^{-\frac{i\hat{V}\Delta t}{2\hbar }}F^{-1}\left(e^{-\frac{i\hat{K}\Delta t}{\hbar }}F\left(e^{-\frac{i\hat{V}\Delta t}{2\hbar }\psi \left(x,t\right)}\right)\right)$$

Where $\hat{V}$ and $\hat{K}$ are the potential and kinetic energy operators, and F and $F^{-1}$ represent Fourier transform and its inverse. From this, shape of the wavefunction at each time step can be assessed, where the holes are coming to the surface in *z* direction; thus, the average potential on x-y plane can be used to calculate the hole flux at each time step. The time step Δt is in femtoseconds, which captures the charge carrier dynamics and provides electronic level insights. The hole flux j defined as the speed of holes at each time step and at each location z is calculated according to equation (23).

(23) $$j\left(z,t\right)=\frac{i\hbar }{2m}\left(\psi \frac{d\psi^{*}}{dz}-\psi^{*}\frac{d\psi }{dz}\right)$$

And summing up the flux over all time steps provides the transmission probability of holes transporting through the material, i.e., providing the current. The absorption spectrum is dependent on the species in each intermediate step, and the photocurrent depends on the probability of holes reaching the surface of each intermediate according to equation (24). The number of absorbed photons (I) depends on the wavelength-dependent solar spectrum S(*λ*), absorption coefficient $\alpha \left(\lambda \right)$ that can be obtained from GW-BSE calculation, and the transmission probability for holes *ϕ* (z) to reach the surface.

(24) $$I=\overset{d}{\underset{0}{\int }}\phi \left(z\right)\left(\overset{\infty }{\underset{0}{\int }}S\left(\lambda \right)\alpha \left(\lambda \right)e^{-\alpha \left(\lambda \right)z}d\lambda \right)dz$$

**Figure 7:**
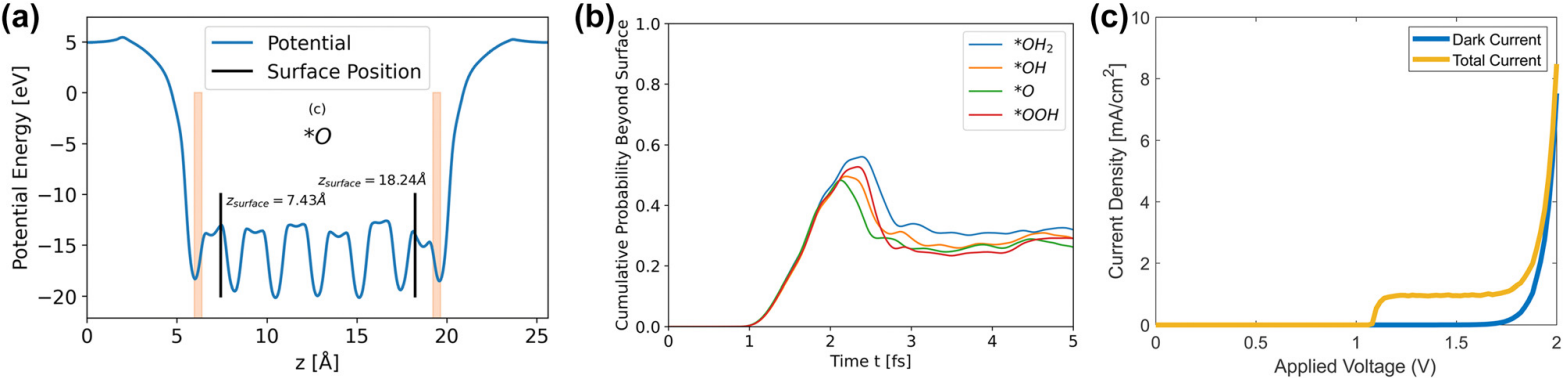
Wavepropagation approach linking the rate determining step to photocurrent. (a) Potential energy variation from bulk to surface in *O reaction intermediate. (b) Maximum probability of hole flux beyond the surface and (c) simulated current density–voltage (J–V) curve with photocurrent from wave propagation method. Adapted from [38]. Copyright 2024, Royal Society of Chemistry, CC BY-NC 3.0 Unported Licence.

By including charge recombination effects in equation (24), total hole flux to the surface was calculated. This resulted in simulating the J–V curve with photocurrent. Holes have to reach the surface fast and also avoid recombination to generate photocurrent. The photocurrent is effected by the potential energy of various reaction intermediates as a function of distance along the surface (*z*-direction), for example in intermediate *O shown in Figure 7a. In *O intermediate species, a midgap state appears in its bandgap [17] that reduces the total initial energy, essentially making a low initial kinetic energy. Thus, there is a low probability for the holes to reach the surface [38] as shown in Figure 7b. Hence, the reaction rate of *O is the slowest and practically the bottleneck for OER reaction. Running the full simulation with photocurrent generates the total current, and KMC simulation [35] generates the dark current as shown in current density-bias plot in Figure 7c. The total current density rises at the onset, reaches a plateau, and then rises again when the dark current increases. The plateau stems from the decrease of electron density at the surface n_s_, which at some voltage becomes negligible. The current density increases due to dark current is near 1.8 V. The increase in total current density at the onset potential in the simulation is about 1.2 V, which is in agreement with experiments [38], [43].

## 5 Employing theoretical methods from modeling water oxidation to study CO_2_ reduction

Reducing the harmful CO_2_ gas into useful fuels such as CH_3_OH and CH_4_ is widely studied , but the first challenging step is the conversion of CO_2_ into CO, which involves two electrons similar to four electrons involved in OER reaction. Thus, the same theoretical methods used for absorption spectra studies on Fe_2_O_3_ during oxygen evolution reaction on the anode can be used to understand the CO_2_ reduction reaction (CO2RR) mechanism to form CO at the cathode by calculating the absorption spectrum of individual reaction intermediates, activation barriers, and electron transport.

### 5.1 CO_2_ reduction reaction modeling using DFT

The CO_2_ reduction to CO mechanism involves two electrons and protons [47] as shown in equation (25).

(25) $$CO_{2}+2\;{\text{H}}^{+}+2e^{-}\to CO+H_{2}O$$

This proton-coupled electron transfer reaction has two intermediate reactions. Once the CO_2_ molecule adsorbs on the surface of the catalyst on an exposed active site (*), H^+^ attaches to CO_2_ to form COOH adsorbate, and when the next H^+^ attaches to COOH, CO and H_2_O leave the surface as shown in equations (26)–(29).

(26) $$*+CO_{2}\to {CO_{2}}^{*}$$

(27) $${CO_{2}}^{*}+{\text{H}}^{+}+e^{-}\to COOH^{*}$$

(28) $$COOH^{*}+{\text{H}}^{+}+e^{-}\to CO^{*}+H_{2}O$$

(29) $$CO^{*}\to CO+*$$

**Figure 8:**
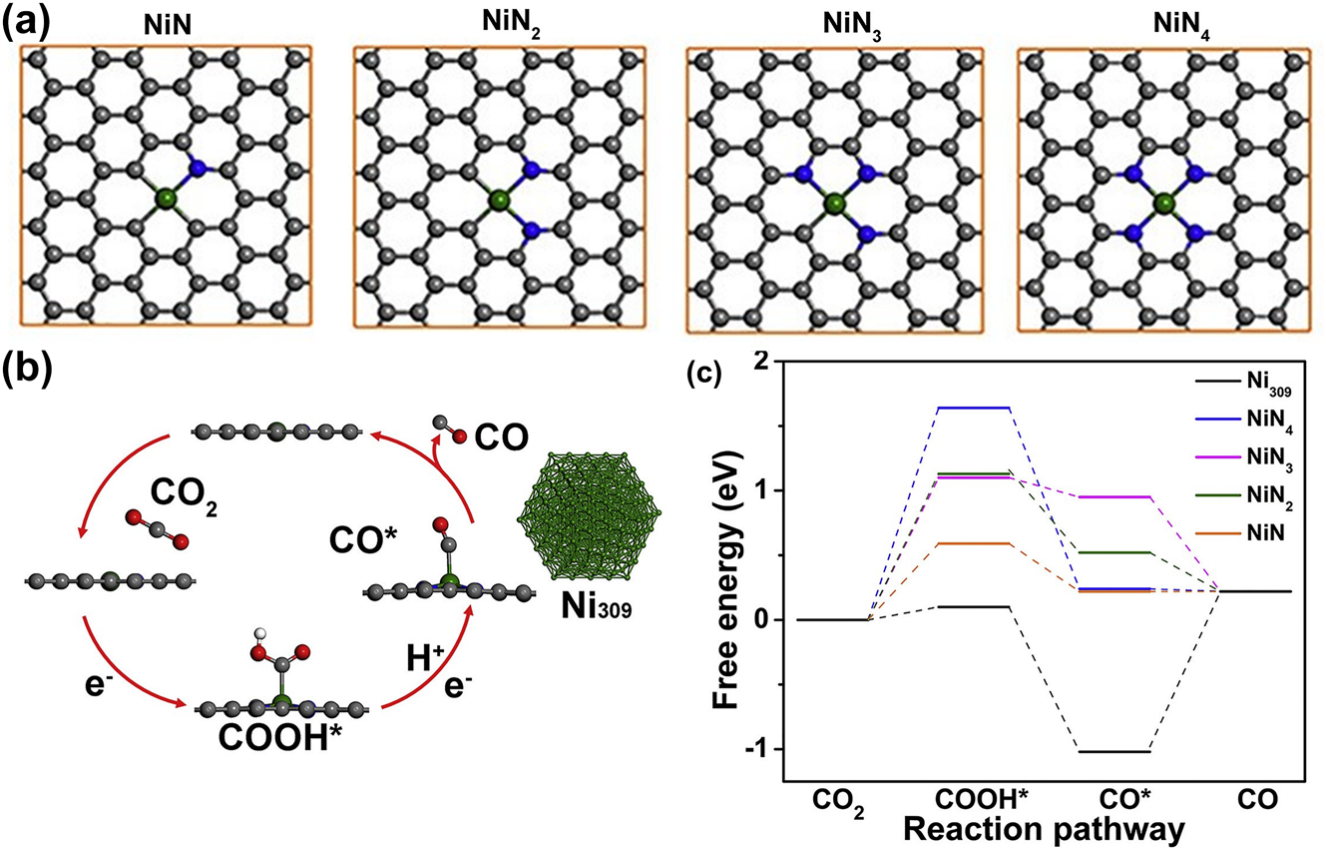
CO_2_ reduction reaction modeling using DFT. (a) Metal-nitrogen-doped carbon (MNCs) with M = Ni (in green) with varying N (in blue) concentration, (b) catalytic cycle for CO_2_ to CO reduction reaction with adsorbates representing the intermediate reactions involved, (c) reaction pathway for these intermediate reactions involving proton-coupled electron transfer for MNC and Ni_309_ cluster. Adapted with permission from [48]. Copyright 2018 Elsevier Inc.

The reaction pathway for the above reactions was studied by Vijay et al. [47] on surface of Au, metal-nitrogen-doped carbon catalysts (MNCs), where M=Fe and Ni. They performed DFT calculations using RPBE exchange correlation functional with Hubbard U parameter of 2 eV added to d orbital of Fe while calculating the Gibbs free energy. Similarly, a comparison of MNC with Ni atoms and Ni_309_ cluster was studied by Zhao et al. [48]. Figure 8a shows the supercells used to model the mechanism of CO_2_ reduction to CO. The schematic of the mechanism and the free energy along the reaction pathway is shown in Figure 8b and c. Replacement of graphene-based catalyst with graphene-like stanene, where Sn atoms forming the hexagonal array, was explored by Giri et al. [49] to find the preferred adsorption site for transition metal (which acts as an active site) on the stanene by considering the two-step reaction pathway mechanism. The CO evolution rate was assessed by calculating the turnover frequency (TOF) as the moles of CO evolved per active site per second on Ni (111) surface [50] using the kinetic Monte Carlo (KMC) approach by using Gibbs free energy obtained from DFT as an input to KMC simulation. As previously seen in the OER reaction of H_2_O, the evolution rate depends on the surface coverage and reaction mechanism that depends on the rate-limiting intermediate specie. This shows that along with the chosen material, since adsorption site influences the Gibbs free energy, it will have an effect on the catalytic site interaction and surface coverage thus will influence the CO evolution rate.

### 5.2 Absorption spectra

**Figure 9:**
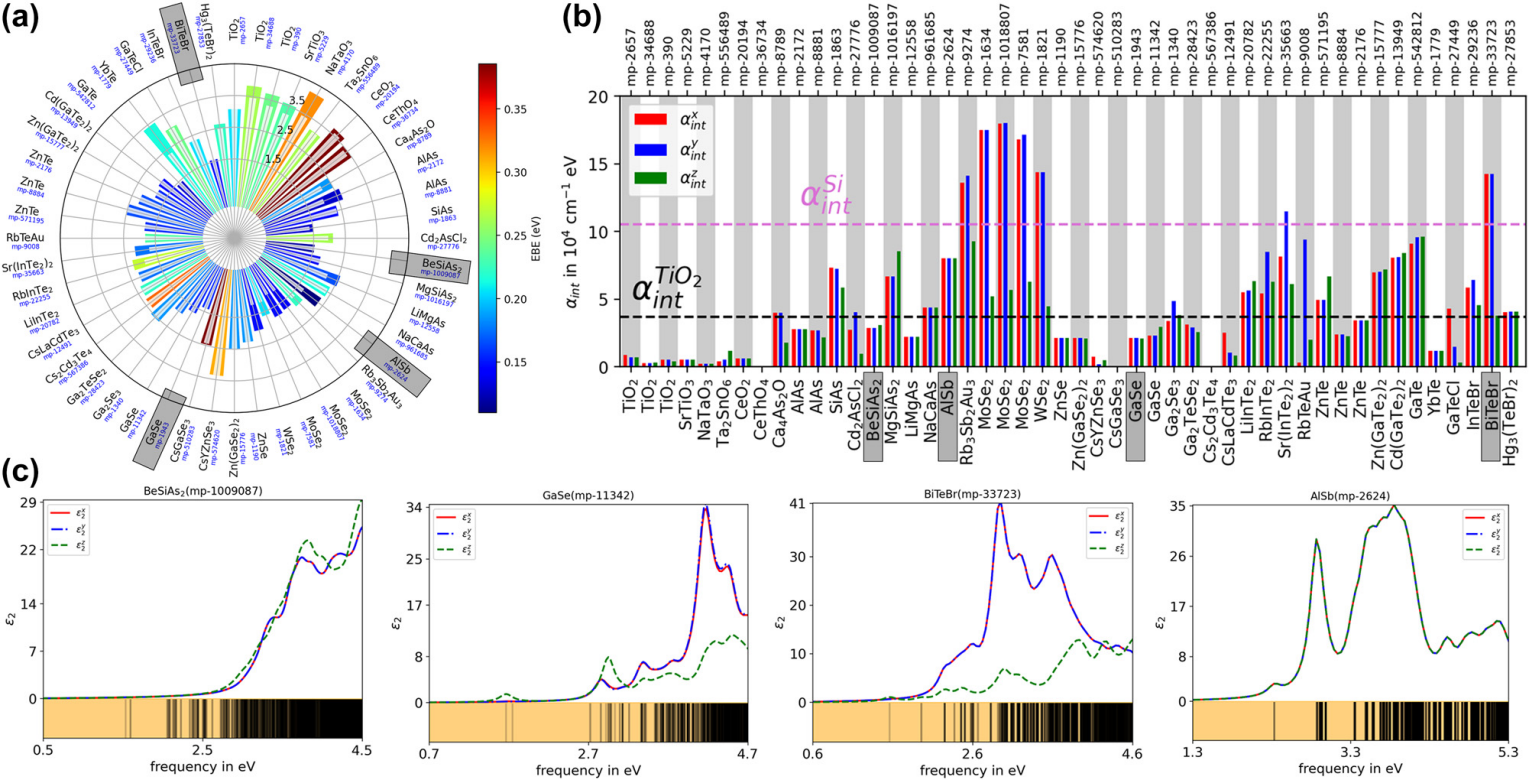
Optical absorption for CO_2_ reduction reaction. (a) Electronic binding energy of various bulk materials for CO_2_ reduction reaction. (b) Absorption coefficient of these materials. (c) Dielectric constant of four chosen materials highlighted in gray rectangles in Fig a and b capturing lower, intermediate, and higher electronic binding energies (EBE) and absorption coefficient (*α*). Adapted from [51]. Copyright 2021, Nature Publishing Group, CC-BY 4.0.

Similar to absorption spectra studies for OER, CO_2_ reduction reaction mechanism can be understood by calculating the absorption spectra of the photocatalyst. Biswas et al. [51] generated optical absorption spectra of nearly 50 photocatalysts using the GW-BSE approach and also related the spectra to the electronic binding energy to find promising photocatalysts for CO_2_ reduction. From Figure 9a and b, we see that the electronic binding energy (EBE) has an impact on the absorption coefficient. The EBE varies from 1.5–3.5 eV, and even a slight variation in EBE affects the absorption coefficient α. For four chosen materials, BeSiAs_2_ and GaSe have absorption coefficient lower than the 4 × 10^−4^ cm^−1^ eV, BiTeBr has greater than 11 × 10^−4^ cm^−1^ eV, and AlSb is in the intermediate range. In all these four materials, the variation in EBE is only 1 eV. The dielectric constants of these materials also showed variation due to changes in electronic binding energy and absorption coefficient as can be seen in Figure 9c. It is to be noted that in these materials, only the bulk absorption properties were calculated; however, the CO_2_ reduction happens on a particular facet. That facet needs to be explored, and the actual absorption coefficient needs to be measured in the presence of CO_2_ in an electrolyte solution.

While most literature focuses on bulk absorption, some on understanding the formation energies and kinetics of two-step proton coupled electron transfer process but not on the absorption spectra of individual reaction intermediates. Thus, GW-BSE method used to generate absorption spectra of individual reaction intermediates during water splitting can be used also for the CO_2_ reduction mechanism. Furthermore, the wavepropagation method can be used to study CO_2_ reduction reaction. We can study how the potential varies for each intermediate reaction and at what voltages this reaction will be activated. The contribution from dark current and photocurrent can be delineated and the onset potential can be calculated. Since in reduction reaction, electrons are involved, electron flux needs to be considered rather than the hole flux that was used for the oxidation reaction.

## 6 Conclusions

Since oxygen evolution reaction can be facilitated by light–matter interaction, the reaction mechanism can be better understood by analyzing the photoabsorption spectra of the nanocatalyst on which the reactions occur. Here are the conclusions drawn on each theoretical method for calculating a specific property related to photoelectrocatalysis. First, the Gibbs free energies of individual reaction intermediates helped understand the overpotential and served as an input to understand kinetic properties. The GW-BSE based on an initial DFT solution helped generate the photoabsorption spectrum and the exciton strength for each reaction intermediate. Second, a combination of Marcus theory with the climbing image nudged elastic band method helped identify the kinetic barriers of the surface intermediate reactions. Finally, the current–voltage (I–V) plots can be generated using the wave propagation approach. A combination of these approaches concludes that *O reaction intermediate is the reactant of the rate limiting step for OER on hematite. As a result, the surface in most of the operation time is occupied with this intermediate.

Thus, the combination of these techniques helped to delineate that the *O intermediate is the rate limiting step in agreement with experimental data. Such insights from computational tools can aid in improving efficiency by providing insight on how to carefully tune experimental conditions and material composition. The example of Fe_2_O_3_ presented here serves as a test case for resolving the absorption spectra for water splitting under photon illumination.

Finally, reducing CO_2_ to useful chemicals involves several reaction pathways, and finding the rate-determining step is helpful. The understanding of absorption spectra, surface coverage by several reaction intermediates, and charge transfer toward their surfaces is essential to understand the mechanism involved. Thus, a combination of these methods, which were used to understand water oxidation reaction, can be applied to understand mechanisms involved in CO_2_ reduction reaction.
